# Phenotypic Characterization of Five Children With PACS1‐NDD: Longitudinal Insights Into Development, Behavior, and Brain

**DOI:** 10.1111/cge.70204

**Published:** 2026-07-02

**Authors:** Fiona Journal, Nada Kojovic, Kenza Latrèche, Stefania Solazzo, Marie Schaer

**Affiliations:** ^1^ Department of Psychiatry, Faculty of Medicine University of Geneva Geneva Switzerland; ^2^ Faculty of Psychology and Science of Education (FAPSE) University of Geneva Geneva Switzerland; ^3^ Fondation Pôle Autisme Geneva Switzerland

**Keywords:** ADHD, ASD, developmental trajectories, eye‐tracking, MRI, PACS1 neurodevelopmental disorder, Schuurs–Hoeijmakers syndrome

## Abstract

PACS1 neurodevelopmental disorder (PACS1‐NDD), also known as Schuurs–Hoeijmakers syndrome, is a rare genetic condition caused by a recurrent de novo mutation in the PACS1 gene. Autistic traits have been reported in PACS1‐NDD, but systematic longitudinal assessments are lacking. We followed five children (3 females) with genetically confirmed PACS1‐NDD, aged 1.4–6.2 years at entry, over 2–3.5 years (29 assessments). Measures included standardized behavioral and cognitive assessments, eye‐tracking, and structural MRI. Data were compared to 357 autistic and 123 typically developing children from a longitudinal cohort. Children with PACS1‐NDD showed global developmental delays with heterogeneous trajectories. Adaptive and communication profiles overlapped with autistic children, while motor impairments were more pronounced. Vocabulary and grammar were delayed, but pragmatic skills were relatively preserved. All children with PACS1‐NDD exhibited autistic traits with elevated restricted and repetitive behaviors and milder social‐communication difficulties. ADHD symptoms were subthreshold and predominantly inattentive. Eye‐tracking revealed preserved social interest but reduced gaze typicality in naturalistic contexts. MRI showed globally reduced gray and white matter volumes. These findings provide the first longitudinal, multimodal characterization of PACS1‐NDD, informing clinical care and targeted outcome measures for therapeutic trials, and highlighting the need for larger studies to validate and extend these findings.

## Graphical abstract

Longitudinal multimodal assessment of five children with PACS1‐NDD revealed global developmental delays, prominent restricted and repetitive behaviors, relatively preserved social interest, heterogeneous language trajectories, and reduced gray and white matter volumes. These findings provide candidate outcome measures for future therapeutic trials.

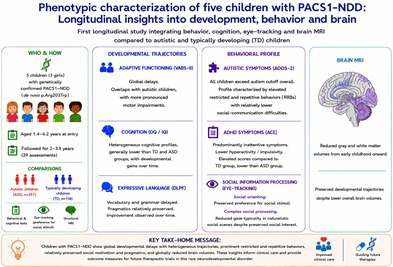

## 1 Introduction

PACS1 neurodevelopmental disorder (PACS1‐NDD), or Schuurs–Hoeijmakers syndrome, is a rare autosomal dominant condition caused by a mutation in the PACS1 gene [1]. PACS1 plays a critical role in cellular homeostasis, particularly regulating membrane trafficking [2]. The syndrome often includes characteristic facial morphology and congenital anomalies affecting the brain, heart, eyes, genitourinary, and skeletal systems [3, 4, 5]. Hypotonia is common in infancy [6] and seizures are frequent, though generally well controlled with medication [6, 7, 8, 9]. Neuroimaging shows brain abnormalities in 65% of individuals, most commonly hypoplasia or partial agenesis of the cerebellar vermis [10]; microcephaly occurs in 20% [11]. Most individuals exhibit moderate to severe developmental delay or intellectual disability, and many display features consistent with autism spectrum disorder (ASD) [6, 7, 9]. Behavioral difficulties, including emotional dysregulation, are also described [6, 7, 12, 13], suggesting impairments in executive functioning.

The genetic homogeneity of PACS1‐NDD makes it an attractive target for precision medicine [14]. As therapeutic possibilities rapidly emerge [15], sensitive and clinically meaningful outcome measures are critical for trials. Although nearly 200 individuals with PACS1‐NDD have been described [6, 7, 9, 11, 16, 17], systematic characterization of neurodevelopmental impairments remains limited. Few studies provide precise cognitive functioning [17, 18], and no prior work has applied validated state‐of‐the‐art diagnostic tools to assess ASD, executive functions, or Attention Deficit with or without Hyperactivity Disorder (ADHD). Furthermore, no study to our knowledge has reported longitudinal measures in PACS1‐NDD.

Families of children with PACS1‐NDD face challenges in accessing appropriate healthcare and therapies due to the syndrome's rarity and recent recognition. It is often absent from standard diagnostic panels, clinical care pathways, and insurance coverage, increasing caregiver burden and limiting access to tailored interventions. Detailed characterization of the PACS1‐NDD and its impact on families is therefore essential.

Here, we applied a comprehensive, autism‐focused deep phenotyping pipeline to examine functional impairments in PACS1‐NDD. We longitudinally assessed five children (3 females; 1.4–9 years old) using standardized cognitive, adaptive, and language measures. Developmental trajectories were compared to 123 typically developing (TD) peers and 357 children with ASD from our longitudinal cohort [19, 20, 21, 22]. Gold‐standard assessments were applied for ASD and ADHD. Eye‐tracking paradigms assessed social cognition, and structural MRI provided a preliminary index of brain‐level characteristics. By integrating clinical, behavioral, eye‐tracking, and neuroimaging data, this study offers a first longitudinal, multimodal characterization of PACS1‐NDD, highlighting shared and syndrome‐specific pathways of atypical development. Based on previous clinical descriptions of PACS1‐NDD and reports of autistic features, developmental delay, and neuroimaging abnormalities, we expected that children with PACS1‐NDD would show global developmental delays across adaptive and cognitive domains and also language delays with some heterogeneity in line with previous research [6, 7, 9]. We expected to observe both the presence of autistic features and variability in their manifestations and severity. Based on our clinical experience and on a previous study reporting that children with PACS1‐NDD generally enjoy social interactions [6], we hypothesized relatively preserved social motivation and engagement leading to enhanced attention toward social stimuli in eye‐tracking tasks. Finally, in line with previous studies, we expected to find brain abnormalities mainly characterized by white matter abnormalities [6, 10].

## 2 Material and Methods

### 2.1 Participants

#### 2.1.1 PACS1‐NDD

Children with PACS1‐NDD were recruited through the PACS1 family association (https://www.pacs1.org), through word of mouth among families, and through outreach to local geneticists. To be included in the study, children with PACS1‐NDD had to fall within the age range of the autistic and typically developing (TD) participants enrolled in the Geneva Autism Cohort (i.e., 1.5 years to 10 years old), allowing meaningful comparisons across groups. In addition, families needed to be proficient in either English or French and able to travel to Geneva for the main longitudinal visits, as the assessments were conducted in‐person. While the protocol included assessments every 6 months, families were required to travel onsite for the yearly visits; intermediate assessments, consisting of solely parental interviews and questionnaires, could be conducted remotely.

Among the five children with PACS1‐NDD, age at first assessment ranged from 1.4 to 6.2 years. All participants carried the recurrent heterozygous PACS1 variant c.607C>T (p.Arg203Trp), a missense mutation identified through clinical diagnostic next‐generation sequencing approaches (whole‐exome sequencing or targeted panels depending on the case). Segregation analysis indicated a de novo occurrence for all five participants. Children completed 5–7 visits (29 total assessments), spanning ages 1.4 to 10.0 years (Figure S1), with a mean follow‐up duration of 2.9 years. Assessments were conducted every 6 months during the first 2 years and annually thereafter. For families living far from the research center (4/5), interim 6‐month assessments were conducted remotely and included parent‐reported measures only. Medical characteristics are provided in Table S1.

#### 2.1.2 ASD and TD Comparison Samples

Comparison data were drawn from the Geneva Autism Cohort, a longitudinal study of developmental trajectories of autistic children [19, 20, 21, 22]. This dataset included 357 autistic (1376 visits, 64 females) and 123 TD children (388 visits, 52 females) aged 0.78 to 10.0 years old. TD participants had no developmental or neurological conditions and no first‐degree relatives with ASD. Most children (80%) contributed at least two visits; over half (56%) had four or more visits. Analyses were conducted on subsamples based on data availability. Detailed descriptions of the ASD and TD cohorts, including recruitment procedures, diagnostic characterization, and developmental outcomes, have been reported elsewhere [19, 22]. In the present study, these data were used exclusively as reference groups to contextualize developmental trajectories observed in children with PACS1‐NDD.

## 3 Measures and Analyses

### 3.1 Behavioral Assessments

#### 3.1.1 Adaptive Functioning

Adaptive functioning was measured using the Vineland Adaptive Behavior Scales, 2nd edition (VABS‐II) [23], a semi‐structured parent interview evaluating communication, daily living skills, socialization, and motor skills. Data were available for all PACS1‐NDD visits and for both comparison groups (*N* = 357 ASD, 1376 visits; *N* = 123 TD, 388 visits).

#### 3.1.2 Cognitive Functioning

In the PACS1‐NDD group, cognitive functioning was evaluated across 22 visits using age‐appropriate standardized instruments (MSEL [24], WPPSI‐IV [25], WISC‐V [26]). The MSEL (used for 19 visits) was administered to children up to 5.7 years old in line with the scale norms. For three of these 19 visits, the child's age exceeded 5.7 years, but their ability to understand complex task instructions was judged insufficient for the WPPSI‐IV or WISC‐V, and the MSEL was therefore administered instead. For the three remaining visits, the WPPSI‐IV and WISC‐V were administered as the children's ages were above 5.7 years and they presented with sufficient comprehension of complex task instructions. For MSEL assessments, DQ scores were computed as developmental age divided by chronological age. For Wechsler scales (WPPSI‐IV and WISC‐V), IQ measures were used. This approach allowed cognitive functioning to be expressed using developmentally appropriate metrics across age periods while facilitating longitudinal characterization of cognitive trajectories.

In the ASD and TD comparison samples, cognitive functioning was evaluated using the same age‐appropriate standardized instruments (MSEL, WPPSI‐IV, WISC‐V). Since the MSEL was introduced later in the cohort (but before the inclusion of participants with PACS1‐NDD), a portion of autistic and TD participants were assessed with the PEP‐3 [27], a developmental assessment similar to the MSEL, for which we computed DQ scores in the same manner. The comparison groups were composed of 357 autistic children (1339 visits; 1124 MSEL, 112 PEP‐3; 99 WISC‐V; 4 WPPSI‐IV) and 123 TD children (380 visits; 269 MSEL; 64 WISC‐V; 42 PEP‐3; 5 WPPSI‐IV).

#### 3.1.3 Language

Expressive language development was assessed using the *Développement du Langage Productif en Français* (DLPF) [28], a parent‐report instrument measuring vocabulary, grammar, and pragmatics in French‐speaking children aged 18–42 months. Analyses were informed by previously defined language profiles in autism (language unimpaired, language impaired, and minimally verbal) [19] and compared to 82 TD children (186 visits).

#### 3.1.4 Developmental Trajectories

Trajectories of adaptive functioning, cognition, and language were examined using linear mixed‐effects models, with age and diagnosis as fixed effects and a within‐subject term modeled as a random effect. Random slope model analyses were carried out using the Mixed Model Trajectories toolbox (publicly available at: https://github.com/danizoeller/myMixed‐ModelsTrajectories) implemented in MATLAB R2019b (MathWorks). Model complexity (constant, linear, quadratic) was selected using the Bayesian information criterion to avoid overfitting [19, 22, 29, 30]. Given the small PACS1‐NDD sample size, analyses were descriptive and no formal statistical testing was performed.

#### 3.1.5 Autistic Symptoms

Autism symptoms were assessed using the Autism Diagnostic Observation Schedule, Second Edition (ADOS‐2) [31], and the Autism Diagnostic Interview‐Revised (ADI‐R) [32]. The ADI‐R evaluates historical symptoms across social interaction, communication, and restricted and repetitive behaviors (RRBs), using established cutoffs (respectively, 10, 8, and 3). The ADOS‐2 provides standardized observational measures with module‐specific thresholds. Analyses focused on descriptive characterization at the individual level. Comparison data were available for 299 children with ASD and 111 TD participants (ADI‐R), and 357 ASD children and 118 typically developing children (ADOS‐2).

#### 3.1.6 Attention

Inattention and Hyperactivity/impulsivity were assessed using the ADHD Child Evaluation (ACE) [33], a structured parent interview for children aged 5–16 years. Diagnostic thresholds require at least 6 out of 9 symptoms in either domain. Four children with PACS1‐NDD were evaluated (one was too young). Comparison included 75 autistic children and 34 TD children.

### 3.2 Eye‐Tracking Derived Measures of Social Information Processing

Eye‐tracking data were acquired using the Tobii TX300 system. Children were seated approximately 60 cm from the display monitor (resolution: 1200 × 1920 pixels; refresh rate: 60 Hz). A five‐point calibration procedure featuring animated, child‐friendly stimuli was implemented via the Tobii system's integrated calibration tool. Social orienting and social cognition were quantified using established paradigms [34, 35].

#### 3.2.1 Social Orienting

Children viewed side‐by‐side dynamic stimuli contrasting social (children in motion) and non‐social (geometric patterns) content [34, 36]. Gaze metrics were analyzed using Tobii Studio (v3.4.8). Social orienting was quantified as the proportion of time spent viewing social stimuli relative to total viewing time [34].

#### 3.2.2 Complex Social Processing

Participants watched a short cartoon (*Trotro* [37]) featuring naturalistic social interactions. Gaze behavior was quantified using the *Proximity Index* (PI), which measures similarity to normative gaze patterns derived from TD children [35]. A normative reference was established using gaze recordings from TD children aged 1.4 to 6.2 years, obtained through our longitudinal study. Analyses included participants with valid data (> 50% viewing time), resulting in reference samples of 93 TD and 233 ASD children. PI scores range from 0 (no overlap with normative attention map) to 1 (perfect alignment).

### 3.3 Anatomical Brain Images Acquisition and Preprocessing

Structural MRI data were acquired in four children with PACS1‐NDD using a 3 T Siemens Magnetom Prisma scanner (Siemens Medical Solutions, Erlangen, Germany) equipped with a 64‐channel head coil, at Campus Biotech in Geneva, Switzerland. Imaging was conducted during natural nocturnal sleep without sedation, following a specific protocol adapted for young autistic children [38, 39]. High‐resolution images were obtained using a 3D T1‐weighted magnetization‐prepared rapid gradient‐echo (MPRAGE) sequence with the following parameters: repetition time (TR) = 2000 ms, echo time (TE) = 2.47 ms, flip angle = 8°, field of view (FoV) = 256 mm, 208 sagittal slices, and 1 mm isotropic voxel resolution. Anatomical 3D images were preprocessed with *Freesurfer* (v7.1, Linux). These procedures are detailed elsewhere [40, 41]. The images were visually inspected for defects in the reconstruction of the white matter and pial surfaces and manually edited, when necessary, by NK. The senior author (MS) validated the final surface reconstruction. When available, individual brain morphometry (cortical gray matter, subcortical gray matter, white matter, and ventricular volumes) was compared to normative developmental trajectories using the BrainChart framework [42].

## 4 Results

### 4.1 Developmental Trajectories of Adaptive Functioning, Cognition, and Language

**FIGURE 1:**
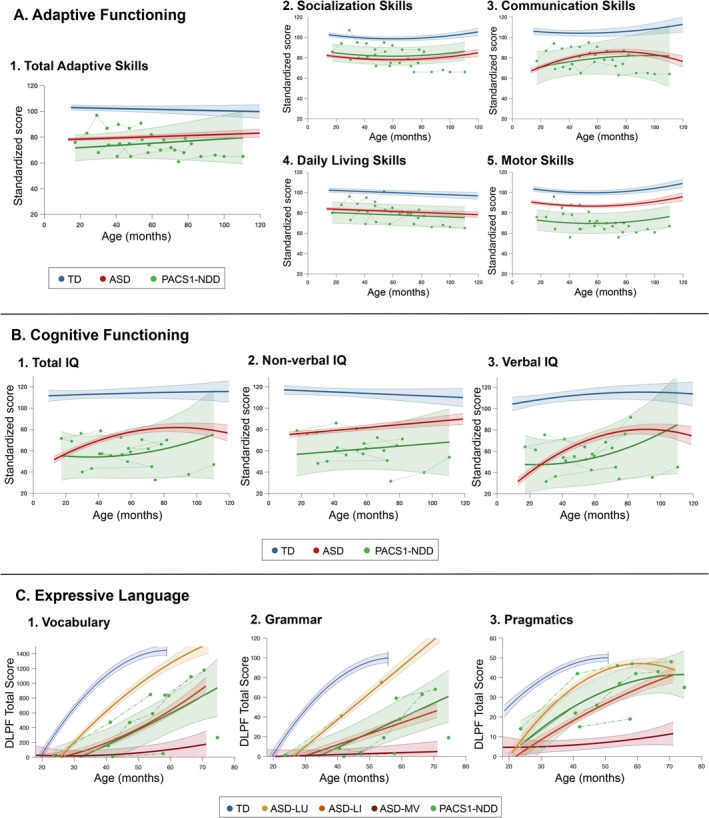
Longitudinal trajectories of (A) adaptive functioning (VABS‐II), (B) cognition, and (C) expressive language trajectories (DLPF total scores) across the three groups of children with PACS1‐NDD, TD, and ASD.

Children with PACS1‐NDD showed lower adaptive skills across all VABS‐II domains (Total, Socialization, Communication, Daily Living Skills, and Motor Skills) than the TD group (Figure 1A). Descriptively, trajectories of children with PACS1‐NDD overlapped with those of children with ASD in most domains (Socialization, Communication, Daily Living Skills), whereas motor skills appeared more impaired in PACS1‐NDD. However, substantial interindividual variability was observed, with some children approaching TD levels. Moreover, adaptive functioning showed a slight increase over development, particularly in Communication and Motor domains (Figure 1 (A3,1A5)), suggesting that children with PACS1‐NDD continue to acquire new skills, and that a subset may partially reduce their delays.

Regarding cognitive functioning, children with PACS1‐NDD scored lower than both TD and ASD groups in total IQ, non‐verbal IQ, and verbal IQ (Figure 1B). PACS1‐NDD IQ trajectories demonstrated an upward trend over time, suggesting developmental progress in verbal and non‐verbal domains. However, trajectories remained heterogeneous across individuals.

Expressive language development was examined longitudinally in four children with PACS1‐NDD (Figure 1C). Three children exhibited trajectories overlapping with autistic children with language impairment (LI), whereas one followed a minimally verbal (MV) profile. On average, first words in PACS1‐NDD emerged around 2.5 years, with approximately 600 words reached by 5 years. Three children reached 800 words by age 5, while two children remained below 100 words by age 5 and below 300 words by age 6. Grammar development showed similar variability, spanning profiles from unimpaired language (LU) to MV. In contrast, pragmatic skills appeared relatively stronger with average trajectories exceeding those of LI and in some cases approaching those of LU.

### 4.2 Autistic Symptoms

**FIGURE 2:**
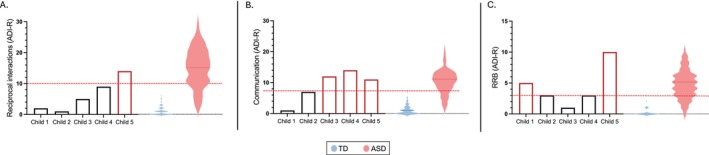
Autism symptoms captured by the ADI‐R. Individual PACS1‐NDD scores are shown on the left, alongside TD and ASD group distributions. Red dotted lines indicate diagnostic thresholds.

As depicted in Figure 2, all five children with PACS1‐NDD presented some degree of autistic symptoms in their developmental history based on parental reports. One child exceeded the clinical cutoffs in all three ADI‐R domains (Reciprocal Social Interaction, Communication, and Restricted and Repetitive Behaviors [RRBs]). Three children exceeded the cutoff in the Social Interaction domain, and two met criteria for RRBs (Figure 2).

**FIGURE 3:**
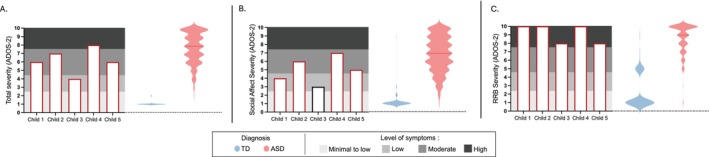
Autism severity captured by the ADOS‐2 across PACS1‐NDD, ASD, and TD groups. Red dotted lines indicate diagnostic thresholds.

Direct assessment using the ADOS‐2 revealed that the PACS1‐NDD group exceeded symptom thresholds (Figure 3). However, symptom distribution across domains was uneven. All children with PACS1‐NDD showed elevated RRB scores, whereas Social Affect (SA) scores ranged from low to moderate.

Item‐level analyses of ADOS‐2 scores show elevated values on items related to RRBs, including hand and finger mannerisms, repetitive interests, and unusual sensory interests for children with PACS1‐NDD (Figure S2). RRB scores were comparable to or even higher than those observed in the ASD group, suggesting prominent non‐social symptoms. In contrast, SA‐related scores were generally lower in the PACS1‐NDD group compared to the ASD group, particularly for joint attention, shared enjoyment, quality of social overtures, and responsive social smile. This pattern suggests a relative dissociation between social and non‐social symptoms in this population, with comparatively milder social difficulties alongside pronounced RRBs.

**FIGURE 4:**
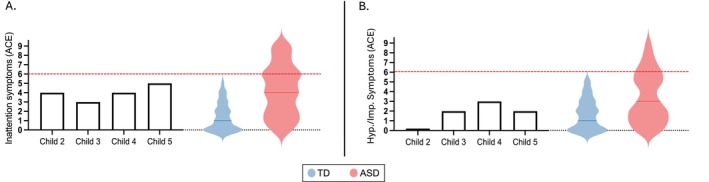
ADHD symptoms assessed with the ACE interview in PACS1‐NDD, ASD, and TD. Red dotted lines indicate diagnostic thresholds.

Regarding ADHD symptomatology, ACE evaluations were available for four children (6.05–9.21 years) (Figure 4). None reached diagnostic thresholds. However, a consistent pattern of greater inattention than hyperactivity/impulsivity was observed.

### 4.3 Eye‐Tracking

**FIGURE 5:**
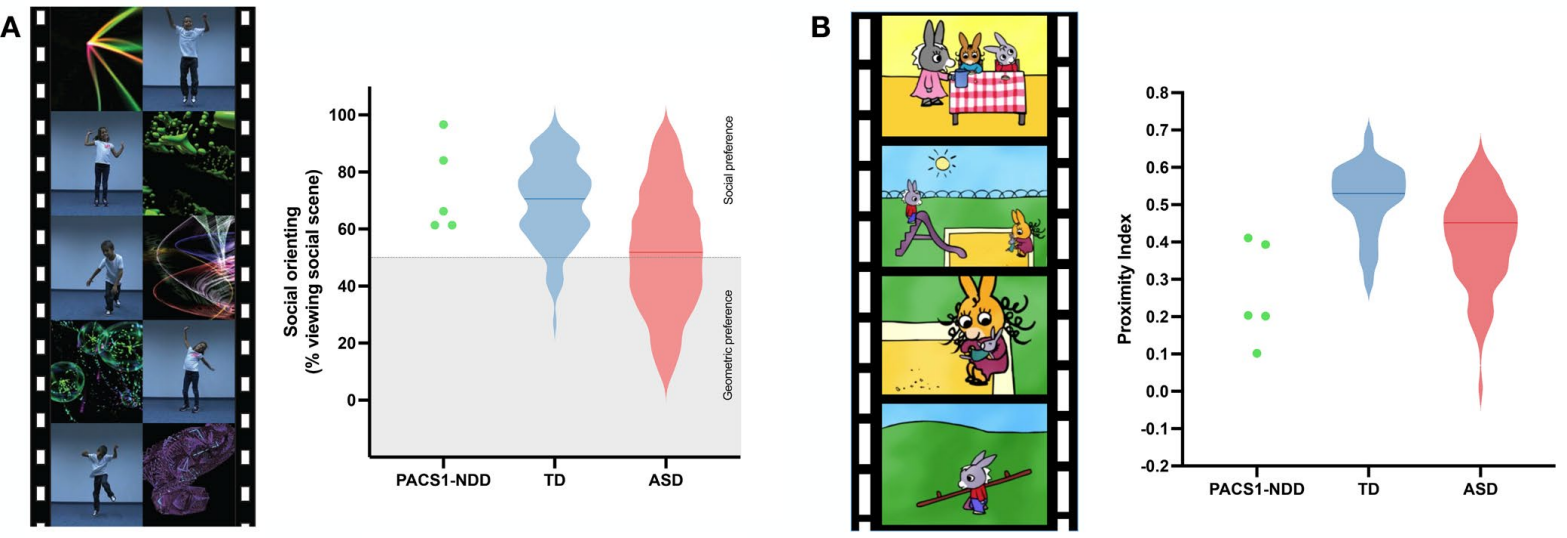
Social information processing assessed using eye‐tracking paradigms: The Social Orienting Task (A) and the Complex Social Processing Task (B).

To investigate social information processing, we employed two eye‐tracking paradigms. In the Social Orienting task, all children with PACS1‐NDD showed a clear preference for social over non‐social stimuli, with values consistently above the 50% threshold (Figure 5A). In contrast, performance in more complex social scenes (cartoon task) showed greater variability. Proximity Index (PI) values ranged from 0.10 to 0.41, compared to averages of 0.52 in TD children and 0.41 in children with ASD (Figure 5B). These findings suggest that, while basic social orienting mechanisms are preserved in children with PACS1‐NDD, challenges may arise in more naturalistic contexts that require the integration of dynamic social cues over time.

### 4.4 Structural Brain Development

**FIGURE 6:**
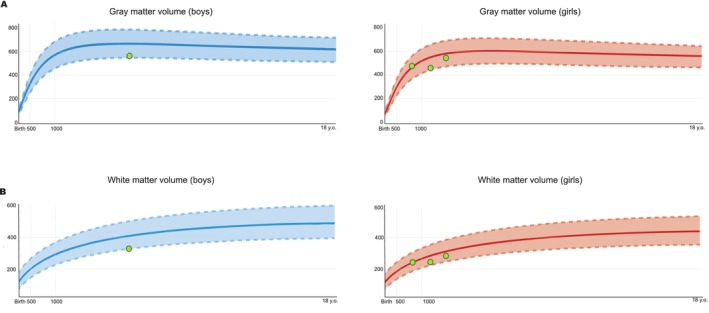
Cerebral gray matter and white matter volume (cm^3^) trajectories in PACS1‐NDD relative to normative growth curves derived from a large reference population [42]. The left panels (in blue) display male trajectories, and the right panels (in red) display female trajectories. The solid lines indicate the normative mean, while the shaded areas denote the 95% confidence interval.

Developmental trajectories of cerebral gray (A) and white (B) matter volumes in children with PACS1‐NDD (circles) are shown in context of normative growth curves derived from a large reference population (Figure 6). The solid lines indicate sex‐specific normative means, and the shaded areas represent the 95% confidence intervals. In both figures, the left panels display data for males and the right panels display data for females.

For cerebral gray matter, children with PACS1‐NDD consistently exhibited volumes below the normative mean across both sexes, though their data points largely fall within the lower portion of the 95% confidence interval. This pattern suggests that, while cortical growth appears to parallel the normative trend, it is reduced in overall magnitude. A similar trend is observed for white matter volume, where children with PACS1‐NDD also showed lower volumes relative to the normative curves. Together, these findings indicate globally reduced gray and white matter volumes from early development, with preserved developmental trajectories.

## 5 Discussion

PACS1‐NDD is a recently identified neurodevelopmental condition that remains insufficiently characterized, particularly in early childhood. Here, we provide a longitudinal and multimodal characterization of five children with PACS1‐NDD followed over 2–3.5 years. Participants were recruited through family associations and word‐of‐mouth, resulting in a convenience sample that may not fully capture the broader phenotypic spectrum of PACS1‐NDD. Nevertheless, the study design enabled detailed characterization of developmental trajectories through 29 repeated assessments, capturing both shared and individual patterns of functioning. The inclusion of deeply phenotyped autistic and typically developing (TD) cohorts further allowed us to contextualize which difficulties may overlap with autism‐related characteristics and which may represent more specific features of PACS1‐NDD. Altogether, this study provides one of the first longitudinal phenotypic descriptions of PACS1‐NDD based on standardized assessments spanning adaptive, cognitive, language, behavioral, and neurobiological domains.

We observed heterogeneous developmental trajectories across children with PACS1‐NDD. Overall, adaptive functioning was consistently lower than in TD children. These deficits highlight the need to support everyday functioning to promote autonomy. Daily living skills trajectories of children with PACS1‐NDD tended to stabilize or even decrease with time, suggesting an increased need for support as age‐related demands become more complex. Socialization skills, by contrast, appeared slightly higher in PACS1‐NDD than in autistic children. Communication trajectories showed gradual improvement in PACS1‐NDD and autism, but remained overall delayed, consistent with previous descriptions of language impairments in PACS1‐NDD [1, 6, 7, 17, 18, 43, 44, 45]. Motor impairments, particularly in fine motor skills, were prominent compared to ASD, aligning with previous studies reporting motor delays [6, 7, 14, 18, 45]. Such motor deficits may impact both daily functioning and performance on cognitively demanding tasks requiring manual responses.

Overall, the five children included in our cohort presented the core clinical characteristics commonly described in PACS1‐NDD, including global developmental delay, hypotonia, language impairment, motor difficulties, autistic traits, and elevated restricted and repetitive behaviors, supporting the clinical relevance of this sample in relation to previously reported cohorts6,7,17. However, although previous reports described greater language impairments compared to motor challenges [6, 14], our PACS1‐NDD sample suggests an opposite pattern, with more pronounced motor difficulties. This discrepancy may indicate that PACS1‐NDD is more phenotypically heterogeneous than previously recognized, despite the recurrent nature of the p.Arg203Trp mutation. In addition, the five children received speech therapy, which may have partially reduced the severity of language difficulties and contributed to this difference. Fine‐grained analyses of expressive language revealed that vocabulary and grammar trajectories resembled those of autistic children with language impairment [19], with some children following minimally verbal profiles. In contrast, pragmatic skills were relatively preserved, consistent with the overall pattern of stronger social functioning. Together, these findings indicate that children with PACS1‐NDD require support across multiple domains, including motor, adaptive, and language skills, while highlighting relative strengths in social communication. The marked interindividual variability observed across children further underscores the importance of individualized interventions and suggests that additional genetic or environmental factors contribute to phenotypic expression.

Our use of gold‐standard diagnostic instruments provides a more nuanced characterization of autistic symptomatology in PACS1‐NDD. The five children with PACS1‐NDD exhibited autistic symptoms based on parental reports, but only one met full criteria across all domains (Social interaction, Communication, and RRBs). Direct assessment with the ADOS‐2 indicated that all five children exceeded the overall autism threshold. However, item‐level analyses suggested relatively higher RRB scores compared to social affect scores [17]. This pattern aligns with the relatively stronger adaptive socialization and pragmatic language skills observed in our sample and may suggest a tendency toward increased rigidity‐related behaviors within this small cohort, although these findings should be interpreted cautiously given the small sample size and the known phenotypic variability of the syndrome.

Importantly, RRBs constitute a highly heterogeneous class of behaviors encompassing not only rigidity and insistence on sameness but also repetitive motor behaviors and unusual sensory interests. Although such behaviors are generally more prevalent and pronounced in autistic populations, they are not specific to ASD [46]. Item‐level analyses suggested elevated repetitive motor behaviors in PACS1‐NDD, including mannerisms and unusual body postures, which may partly relate to the motor difficulties frequently reported in this syndrome. Sensory interests were also commonly observed, and expressive rigidity often co‐occurred with behavioral rigidity. These findings highlight the multidimensional nature of RRBs and suggest that further studies in larger cohorts are needed to clarify which RRB dimensions are most characteristic of PACS1‐NDD and how they relate to broader neurodevelopmental features, including executive functioning difficulties and potential later comorbidities such as ADHD.

Existing reports provide descriptions of behaviors related to ADHD, including behavioral problems [7], hyperactive or impulsive behaviors [6], emotional regulation difficulties [13]. In this work, none of the children with PACS1‐NDD exceeded the ADHD diagnostic threshold, but subthreshold symptoms (predominantly inattention) were consistently observed. This pattern is clinically relevant, as inattentive symptoms are often underrecognized yet tend to persist across development [47, 48, 49]. The presence of motor difficulties in PACS1‐NDD may confound the clinical presentation: reduced physical agitation might be due to motor difficulties. Mental agitation, however, could still be present, warranting more detailed assessment to inform care strategies and improve well‐being. Even in the absence of a formal diagnosis, the use of psychostimulant medication may warrant consideration in clinical evaluation.

Beyond detailed behavioral characterization, our study provides initial insights into potential biomarkers of PACS1‐NDD. Eye‐tracking results suggest that basic social orienting in children with PACS1‐NDD is preserved, suggesting intact early‐stage social attention [34]. However, in more complex and dynamic social interactions [35], children with PACS1‐NDD increasingly diverge from normative gaze patterns. This pattern suggests that social difficulties may arise not from reduced social interest, but from challenges in integrating dynamic social information over time [6, 50]. Converging evidence from ADOS‐2 item‐level analyses supports this view: children performed relatively well on simpler social behaviors (e.g., pointing, shared enjoyment, response to name), but showed greater difficulties in tasks requiring sustained, reciprocal, and integrated social engagement.

These findings suggest that intervention should not only support social motivation, but also enhance the processing and integration of dynamic social cues [51, 52].

Brain imaging was conducted with a night‐time recording protocol without sedation [38, 39]. Across participants with PACS1‐NDD, we observed globally reduced gray and white matter volumes relative to normative populations, across both sexes, consistent with previous studies [6, 53, 54]. This pattern of reduced brain volume may contribute to the cognitive, psychomotor, and attentional difficulties described in PACS1‐NDD, suggesting a diffuse neurodevelopmental alteration affecting multiple functional systems.

Despite phenotypic heterogeneity, PACS1‐NDD is caused by a recurrent mutation (p.Arg203Trp) making it an attractive target for precision medicine approaches. Antisense oligonucleotides (ASO) therapies, which selectively target mutant mRNA [14], have shown promise in other monogenic neurodevelopmental disorders. Preclinical studies in PACS1 models indicate that ASO treatment targeting PACS1 or HDAC6 can restore neuronal architecture and synaptic transmission, highlighting the translational potential of this approach [55]. In this context, the detailed longitudinal and multimodal measures provided in this study may serve as valuable outcome metrics for future clinical trials.

Finally, our study provides the most comprehensive longitudinal investigation of PACS1‐NDD to date, integrating behavioral, cognitive, and neurobiological data. Our findings delineate a distinctive developmental profile characterized by prominent restricted and repetitive behaviors, relatively preserved social communication, and inattention symptoms. These findings underscore the importance of fine‐grained, domain‐specific assessment and highlight executive functioning as a potential key mechanism underlying the phenotype.

The main limitation of this study is the small sample size, which limits statistical generalizability and does not capture the full phenotypic spectrum of PACS1‐NDD. Although our cohort was broadly consistent with previously reported PACS1‐NDD phenotypes, some differences were observed, particularly regarding the relative severity of motor versus language impairments. Future work across broader age ranges will be needed to confirm these patterns and refine clinical care. This study is also limited by the lack of systematic investigation of potential additional genetic factors (e.g., secondary variants, copy number variants, or modifier genes), which may contribute to the observed phenotypic variability. Nevertheless, given the rarity of PACS1‐NDD, these findings provide a foundation for future research. Larger longitudinal studies are needed across broader age ranges to confirm these patterns and to further explore additional clinically relevant domains, such as food selectivity, sleep disturbances, and emotional regulation. Such efforts will be essential to refine clinical care and support the development of targeted therapeutic strategies for this rare condition.

## Author Contributions

Conceptualization: F.J., K.L., N.K., S.S., and M.S.; methodology: F.J., K.L., N.K., and M.S.; formal analysis: F.J., K.L., N.K.; writing – original draft preparation: F.J., K.L., N.K., and M.S.; writing – review and editing: F.J., K.L., N.K., S.S., and M.S.; supervision: M.S.; funding acquisition: M.S. All authors have read and agreed to the published version of the manuscript.

## Funding

This study was supported by the National Centre of Competence in Research (NCCR) Synapsy (Grant No. 51NF40‐185897). Swiss National Science Foundation (SNSF) (Grant Nos. #163859, #190084, #202235, #212653 to M.S.), the Fondation privée des Hôpitaux Universitaires de Genève (https://www.fondationhug.org) and by the Fondation Pôle Autisme (https://www.pole‐autisme.ch). The funders were not involved in this study and had no role other than to provide financial support.

## Ethics Statement

This study was approved by the Ethics Committee of the University of Geneva.

## Consent

Informed consent to participate was obtained from all families involved in the study.

## Conflicts of Interest

The authors declare no conflicts of interest.

## Supporting information


**Figure S1:** Distribution of the longitudinal data used in this study. The bottom panel shows the sample of autistic and typically developing children drawn from the Geneva Autism Cohort that is used as a comparison sample for the sample of children with PACS1‐NDD (top panel).
**Figure S2:** ADOS item‐level scores for children in the ASD (red) and PACS1 (green) groups. Bars represent group means for each item, with horizontal error bars indicating the standard error of the mean (SEM). Scores ranged from 0 (no evidence of abnormality) to 3 (clear evidence of abnormality). We included items from the following domains (A: Communication, B: Reciprocal Social Interaction, and D: Restricted and Repetitive Behaviors).
**Table S1:** Clinical and developmental data of five children with PACS1‐NDD.

## Data Availability

The raw data supporting the conclusions of this article will be made available by the authors on a reasonable request.
