# Supplementary material for: Phenotypic Characterization of Five Children With PACS1‐NDD: Longitudinal Insights Into Development, Behavior, and Brain
Source: Clin Genet. 2026 Jul 2;110(3):347–57. doi: 10.1111/cge.70204 (PMC13432291; doi:10.1111/cge.70204)
Supplement: Supplementary file 1 — Figure S1: Distribution of the longitudinal data used in this study. The bottom panel shows the sample of autistic and typically developing children drawn from the Geneva Autism Cohort that is used as a comparison sample for the sample of children with PACS1‐NDD (top panel). Figure S2: ADOS item‐level scores for children in the ASD (red) and PACS1 (green) groups. Bars represent group means for each item, with horizontal error bars indicating the standard error of the mean (SEM). Scores ranged from 0 (no evidence of abnormality) to 3 (clear evidence of abnormality). We included items from the following domains (A: Communication, B: Reciprocal Social Interaction, and D: Restricted and Repetitive Behaviors). Table S1: Clinical and developmental data of five children with PACS1‐NDD. [file CGE-110-347-s001.docx]

# Additional information

#
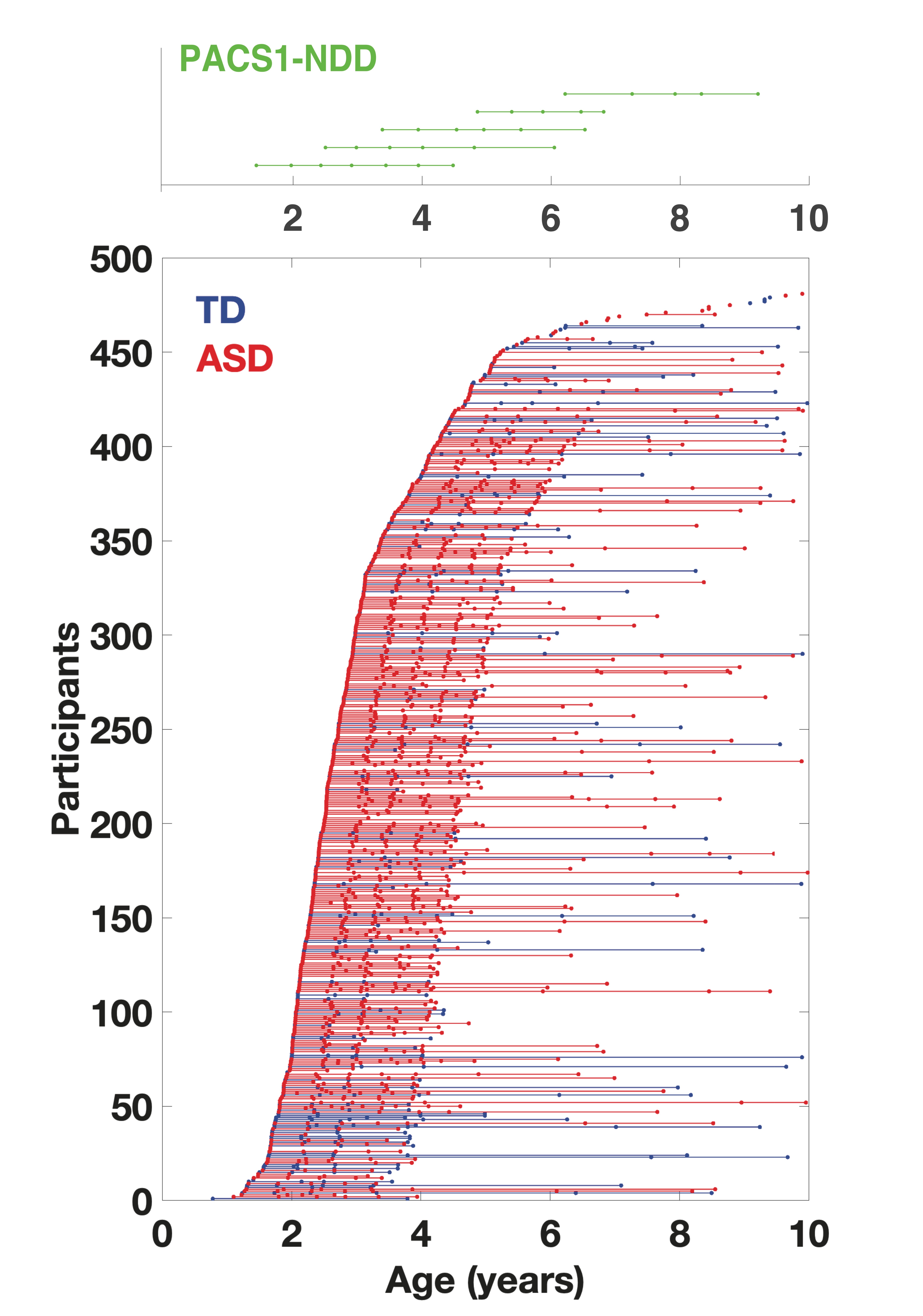


**Figure S1.** Distribution of the longitudinal data used in this study. The bottom panel shows the sample of autistic and typically developing children drawn from the Geneva Autism Cohort that is used as a comparison sample for the sample of children with PACS1-NDD (top panel).


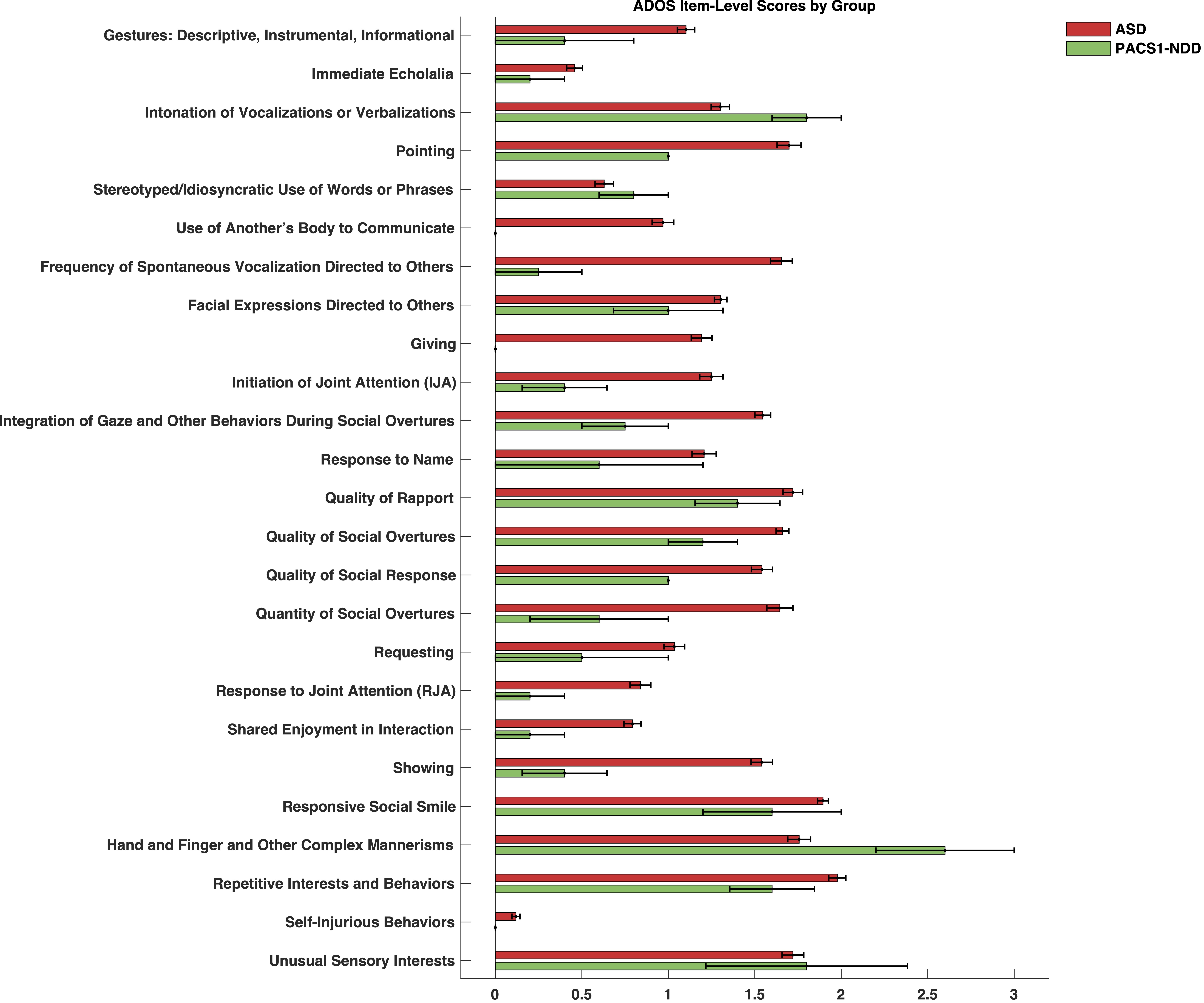


**Figure S2.** ADOS item-level scores for children in the ASD (red) and PACS1 (green) groups. Bars represent group means for each item, with horizontal error bars indicating the standard error of the mean (SEM). Scores ranged from 0 (no evidence of abnormality) to 3 (clear evidence of abnormality). We included items from following domains (A: Communication, B: Reciprocal Social Interaction and D: Restricted and Repetitive Behaviors).

**Table S1.** Clinical and developmental data of five children with PACS1-NDD.

|  | **Child 1** | **Child 2** | **Child 3** | **Child 4** | **Child 5** |
| --- | --- | --- | --- | --- | --- |
| **Ages at visits [years]** |  |  |  |  |  |
| T1 | 1.43 | 2.50 | 3.38 | 4.86 | 6.22 |
| T2 | 1.97 | 2.98 | 3.94 | 5.39 | 7.25 |
| T3 | 2.43 | 3.50 | 4.53 | 5.87 | 7.93 |
| T4 | 2.91 | 4.01 | 4.96 | 6.46 | 8.33 |
| T5 | 3.44 | 4.81 | 5.53 | 6.81 | 9.21 |
| T6 | 3.94 | 6.05 | 6.52 | – | – |
| T7 | 4.48 | – | – | – | – |
| **Genetic findings** | PACS1 c.607C>T (p.Arg203Trp); de novo; targeted NGS panel (Ion Proton platform); Sanger confirmation | PACS1 c.607C>T (p.Arg203Trp); de novo; whole-exome sequencing (Twist capture) | PACS1 c.607C>T (p.Arg203Trp); de novo; whole-exome sequencing (MedExome, Roche); Sanger or qPCR confirmation | PACS1 c.607C>T (p.Arg203Trp); de novo; exome-based identification (detailed report not available) | PACS1 c.607C>T (p.Arg203Trp); de novo; targeted sequencing; Sanger (ABI PRISM 3500XL) |
| **General characteristics** |  |  |  |  |  |
| Biological sex | Female | Female | Female | Male | Male |
| **Birth and growth parameters** |  |  |  |  |  |
| Type of delivery | Vaginal | Elective C-section | Vaginal | Vaginal | Emergency C-section |
| Perinatal complications | Feeding issue (day 3) | Concern for neonatal seizure, excluded | Feeding issue (nasogatric tube) | Feeding issue | Anemia, respiratory + feeding issues (nasogastric tube) |
| Gestational age [weeks+days] |  |  | +6 |  | +1 |
| Birth weight [g] |  |  |  |  |  |
| Height at T1 (percentile) | P50 | P18 | P10 | P18 | P3 |
| Weight at T1 (percentile) | P18 | P25 | P50 | P75 | P3 |
| **Developmental milestones [months]** |  |  |  |  |  |
| Independent walking |  |  |  |  |  |
| First words |  |  |  |  |  |
| First sentences |  |  |  |  |  |
| **Medical findings (past or present)** |  |  |  |  |  |
| Epilepsy | + | – | + | + | – |
| Vision | – | + (hyperopia, astigmatism) | + (hyperopia, astigmatism) | – | – |
| Gastro-intestinal | + | + | + | + | + |
| Sleep problems | + | + | + | – | + |
| Other | – | – | Hyperlaxity, astigmatism, renal cysts, ventricular septal defect | Asthma | Hernia, cardiac malformation, anemia |
| **Behavioral interventions (past or present)** |  |  |  |  |  |
| Speech therapy | + | + | + | + | + |
| Psychomotor therapy | + | + | + | + | + |
| Occupational therapy | – | + | + | + | – |
| Physical therapy | + | – | + | – | – |
| Special education educator | – | + | – | – | + |
| Parental psychotherapy/coaching | – | – | + | + | – |
| Applied Behavior Analysis therapy | – | – | + | + | – |
|  |  |  |  |  |  |
|  |  |  |  |  |  |
|  |  |  |  |  |  |
|  |  |  |  |  |  |
|  |  |  |  |  |  |
|  |  |  |  |  |  |
|  |  |  |  |  |  |
|  |  |  |  |  |  |
|  |  |  |  |  |  |
